# A Generalizable Prioritization Protocol for Climate-Sensitive Zoonotic Diseases

**DOI:** 10.3390/tropicalmed9080188

**Published:** 2024-08-21

**Authors:** Adam C. Castonguay, Sukanta Chowdhury, Ireen Sultana Shanta, Bente Schrijver, Remco Schrijver, Shiyong Wang, Ricardo J. Soares Magalhães

**Affiliations:** 1 Queensland Alliance for One Health Sciences, School of Veterinary Sciences, The University of Queensland, St. Lucia, QLD 4072, Australia; 2 International Centre for Diarrheal Diseases Research, Bangladesh (icddr,b), Dhaka 1213, Bangladesh; sukanta@icddrb.org (S.C.); ireenshanta@icddrb.org (I.S.S.); 3 VetEffect, 3723 BG Bilthoven, The Netherlands; bente.schrijver@veteffect.nl (B.S.); remco.schrijver@veteffect.nl (R.S.); 4 World Bank Group, Washington, DC 20433, USA; swang1@worldbank.org; 5 Children’s Health and Environment Program, UQ Children’s Health Research Centre, The University of Queensland, St. Lucia, QLD 4072, Australia

**Keywords:** climate change, zoonotic disease, disease prioritization, One Health, analytical hierarchy process

## Abstract

Emerging and re-emerging zoonotic diseases pose a significant threat to global health and economic security. This threat is further aggravated by amplifying drivers of change, including climate hazards and landscape alterations induced by climate change. Given the complex relationships between climate change and zoonotic disease health outcomes, a structured decision-making process is required to effectively identify pathogens of greatest concern to prioritize prevention and surveillance efforts. Here, we describe a workshop-based expert elicitation process in six steps to prioritize climate-sensitive zoonoses based on a structured approach to defining criteria for climate sensitivity. Fuzzy analytical hierarchy process methodology is used to analyze data provided by experts across human, animal, and environmental health sectors accounting for uncertainties at different stages of the prioritization process. We also present a new interactive expert elicitation interface that facilitates data collection and real-time visualization of prioritization results. The novel approach presented in this paper offers a generalized platform for prioritizing climate-sensitive zoonoses at a national or regional level. This allows for a structured decision-making support process when allocating limited financial and personnel resources to enhance preparedness and response to zoonotic diseases amplified by climate change.

## 1. Introduction

Previous studies have shown that the majority of emerging infectious diseases and human pandemics in the past century have been the result of zoonotic spillovers, with over 250 zoonoses documented in the literature as newly discovered or rapidly increasing in incidence or geographical range in the past 70 years [1]. Emerging evidence suggests that climate change can exacerbate the impacts of infectious diseases on human and animal health systems. Specifically, recent research suggests that 58% of 375 infectious diseases impacting humanity worldwide have been aggravated by climatic hazards [2]. Moreover, climate change is expected to further increase the transmission of viruses across species over the next decades [3]. Given the large number of infectious diseases that can impact both human and animal populations, and the recognized role of climate change on their distribution and future impacts, decision-support tools are critical for helping countries and/or regions identify priority climate-sensitive zoonotic diseases to help drive investments to ensure effective preparedness and response.

Generally, infectious disease prioritization frameworks consist of four main steps: (1) identify relevant diseases to be ranked, (2) identify criteria to rank diseases, (3) weight criteria using expert judgment, and (4) rank diseases based on the weights attributed to each criterion. The way criteria are weighted has differed across standard methods; for instance, considering criteria as equally important [4], using authors’ opinions and proxies [5,6], Delphi processes and focus group discussions [7,8,9,10,11], utility values derived from conjoint analysis [12,13], probability distribution modeling [14], weighted sum [15,16], and the analytical hierarchy process [17]. This latter approach was used by Rist et al. [18] to develop the One Health Zoonotic Disease Prioritization (OHZDP) Tool designed to facilitate the selection of criteria, questions and answers to score diseases, and weigh criteria based on the approach developed by Saaty [19] using Excel spreadsheets. Several studies have later applied this tool in different regions of the world, adapting criteria, questions, and responses based on their relevance to the country or region of application [20,21,22,23,24,25,26]. Existing prioritization approaches offer a transparent framework that uses a collaborative process, ensuring representative input from stakeholders in both human and animal health sectors. This results in a ranked list of zoonoses based on numerous criteria, which can guide joint efforts in areas of shared interest.

However, the inclusion of climate change impacts on zoonotic transmission in criteria or questions used for prioritizing zoonoses in existing frameworks has remained limited. Few studies have considered climate sensitivity in disease prioritization exercises, assessing the potential impacts of climate change on a disease as a single criterion [14,27,28,29]. The absence of a formal process to capture the level of climate sensitivity of biological processes of transmission and resulting health outcomes is a significant gap in current approaches to zoonotic disease prioritization given growing evidence that climate change is a significant driver of zoonotic disease transmission. Understanding the level of climate sensitivity of a zoonosis will allow disease control managers to recognize which diseases could become an increasing problem under different climate change scenarios relevant to their jurisdictions and which investments in preparedness and response should be undertaken to mitigate their impact. Furthermore, while existing prioritization tools consider variability in expert responses to priority lists of zoonoses, they do not account for the level of confidence of experts when providing their assessment. A formal assessment of uncertainty inherent to expert knowledge across the fields of human, animal, and environmental health is paramount to understanding gaps in zoonotic disease evidence which can help drive investment for research innovation.

In this study, we present a novel six-step approach that directly addresses the shortcomings of current zoonotic disease prioritization frameworks. We first introduce a new process to define the criteria to assess the impacts of climate change on zoonotic transmission dynamics and health outcomes. We then describe the methodological steps leading to the prioritization of climate-sensitive zoonotic diseases using the fuzzy analytical hierarchy process (FAHP). Finally, we present and demonstrate the application of this framework within an online user interface to facilitate the wider implementation of the climate-sensitive zoonotic diseases prioritization protocol by national human, animal, and environmental health experts.

## 2. Materials and Methods

### 2.1. Conceptualizing Climate Sensitivity of a Zoonosis to Develop Criteria for Prioritization

To facilitate the comprehension and communication of the impacts of climate change on health outcomes from zoonotic disease transmission to a wide range of stakeholders and health experts who may not be familiar with these impacts, we developed new criteria for zoonosis prioritization that capture the impacts of climate change.

We started by characterizing climate change by identifying the different climate hazards that can affect ways zoonotic disease transmission in different ways. Based on recent literature reviews, we identified long-term changes in temperature and precipitation, droughts, floods, heat waves, sea level rise, fires, and wind speed as the most important potential hazards resulting from climate change that have been reported to influence zoonotic disease transmission [2,30,31]. 

Second, we identified the different biological mechanisms through which zoonotic transmission can be affected by particular climate hazards based on previous literature reviews [2,32,33,34,35]. These mechanisms include increasing geographic exposure to infected hosts and vectors, change in host or vector population demographics, evolutionary pressure on pathogens, and host susceptibility to infections. 

Each of the biological processes of zoonotic disease transmission described above can affect animal and human health in different ways. To improve our understanding of these linkages, we then reviewed the literature on previous studies that have identified impacts of the particular biological processes of different health outcomes as criteria to inform priorities for the surveillance, prevention, and control of zoonoses. The literature review included all manuscripts and resources published using the Centers for Disease Control’s (CDC) One Health Zoonotic Disease Prioritization (OHZDP) tool [18] applied in a total of twenty-seven countries, with nine papers [21,22,23,24,25,26,36] and fourteen reports, as well as previous studies using different methodologies considering health impact criteria to prioritize pathogens for surveillance [9,16,37,38,39,40]. The resulting list of human and animal health impact criteria was grouped into four commonly re-occurring categories: (1) severity of disease, (2) ability to prevent and control, (3) transmissibility, and (4) socio-economic impacts. 

**Figure 1:**
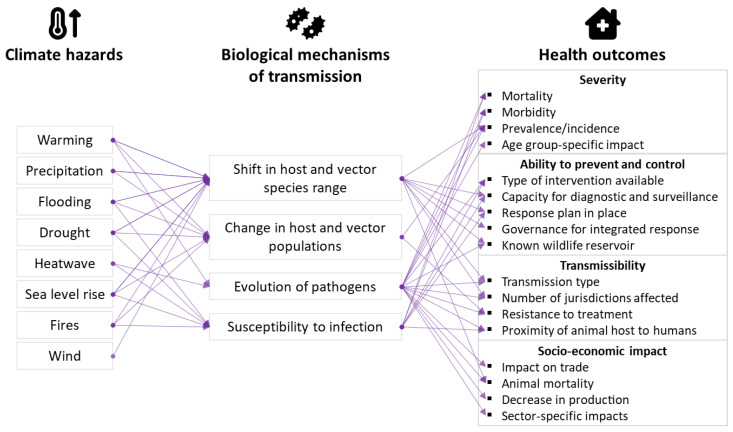
Framework to characterize the multipronged linkages between climate hazards, zoonoses transmission dynamics, and impacts on animal and human populations. Links between domains correspond to available evidence of the relationship between domains.

Based on the three sets of criteria described above, we developed a framework whereby each climatic hazard was linked to biological processes of transmission, which, in turn, was associated with a potential impact on animal and human health (Figure 1). As an example, the climate hazard *drought* is known to have an impact on the *geographic exposure of hosts to vectors and pathogens* (e.g., the distribution of Australian flying foxes), which, in turn, can increase the *prevalence* (severity) of a disease; for instance, in the case of Hendra virus [41]. 

### 2.2. Steps of the Prioritization Process

Based on the framework introduced in Section 2.1, we present the steps of the prioritization process using the Prioritization Tool for Climate-Sensitive Zoonoses (PTCSZ). The process described here follows the FAHP methodology, whereby the knowledge of One Health experts is elicited in a workshop and used to weigh each criterion to enable disease prioritization.

Prior to the workshop, it is critical to collate a preliminary list of zoonoses to be prioritized, criteria to be used in the prioritization, and questions and associated answers for prioritization based on the identified criteria. A master list of pathogens that are potentially sensitive to different climate hazards can be gathered through consultation with local One Health and climate change experts and a literature review of global (e.g., [34,42,43,44]) and local studies on the linkages between climate variables and mechanisms of zoonotic disease transmission. 

**Figure 2:**
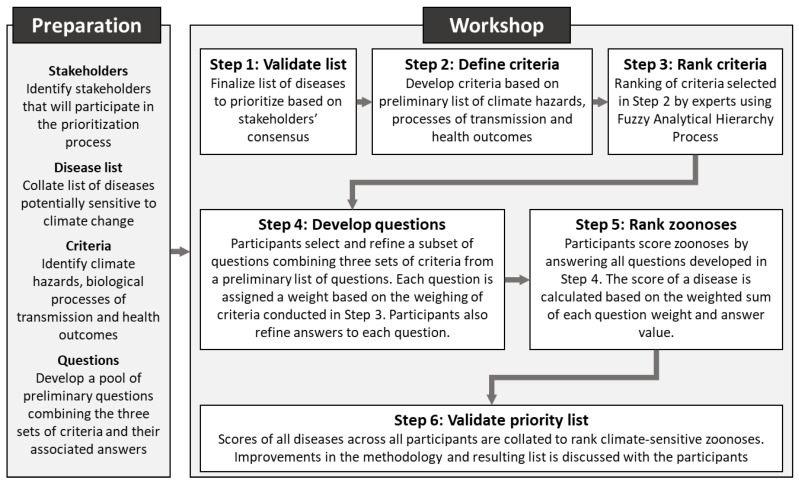
Steps of the prioritization process using the Prioritization Tool for Climate-Sensitive Zoonoses.

The prioritization workshop consists of six steps: (1) validation of a broad list of zoonotic diseases, (2) refinement of climate sensitivity criteria, (3) ranking of criteria, (4) refinement of climate sensitivity questions and answers to be used to score each zoonotic disease, (5) ranking of climate-sensitive zoonoses, and (6) validation of the priority list of climate-sensitive zoonoses (Figure 2).

#### 2.2.1. Workshop Preparation

The preparation for the workshop entails careful identification and invitation of key participants from various One Health sectors, encompassing human, animal, and environmental/wildlife health. The number of voting stakeholders is expected to range from 6 and 15, balancing the objectives of efficient time management within the workshop’s allocated time and resource constraints while ensuring comprehensive representation across sectors [45]. Stakeholders should be chosen from a mix of government agencies and relevant organizations, with considerations for their years of experience, level of expertise, and specific contributions to ongoing national or regional zoonotic disease initiatives. Given the complexity of the nature of climate change and zoonotic disease transmission, it is crucial to ensure that the voting stakeholders, as a group, possess comprehensive knowledge in a range of fields, such as virology, bacteriology, parasitology, immunology, epidemiology, and economics, among others, to make informed evaluations. Additionally, an equitable number of participants from each sector should be included to maintain balanced representation. The selected experts should not only possess deep expertise in their respective fields but also demonstrate a strong commitment to advocating for the adoption of the finalized prioritized list in upcoming prevention and surveillance endeavors within their jurisdictions.

A preliminary list of zoonotic diseases of jurisdictional importance and potentially sensitive to climate change should also be collated at this stage. The list can be created based on expert elicitation prior to the workshop and a review of the literature. A preliminary list of criteria is also prepared at this step based on climate hazards, processes of transmission, and health outcomes outlined in Section 2.1 that are relevant to the case study area. Finally, questions used to score diseases should be developed, whereby the three types of criteria presented in Section 2.1 are combined to capture the multiple linkages between climate hazards and health outcomes. For each question, a set of ordinal answers is formulated following the methodology presented by Rist et al. [18].

#### 2.2.2. Activity 1: List of Zoonotic Diseases to Be Targeted for Prioritization

At the start of the workshop, the first step consists of refining and validating the list of zoonotic diseases to be scored. Since there may be limited data available on the sensitivity of some zoonoses to climate change, the removal of diseases on the grounds of insufficient sensitivity to climate change should be carefully considered to avoid biases associated with the preferences or opinions of the voting members. The refinement of the list can entail the removal of diseases that are not relevant to the country or deemed insufficiently sensitive to climate change. Conversely, additional zoonoses that were not a priori considered can be added to the final list. Diseases that are not yet of concern but that could become a greater threat under climate change should be considered for scoring.

#### 2.2.3. Activity 2: Selection of Climate Sensitivity Criteria

The list of criteria (climate hazards, processes of transmission, and health outcomes) is then refined based on the knowledge and opinions of expert participants. Importantly, a subset of climate hazards should be selected based on their relevance to the country. Furthermore, the most relevant health outcome criteria should be discussed and selected based on the preliminary list generated prior to the workshop. The selected criteria should then be used to formulate questions in Step 4.

#### 2.2.4. Activity 3: Ranking of Criteria

**Figure 3:**
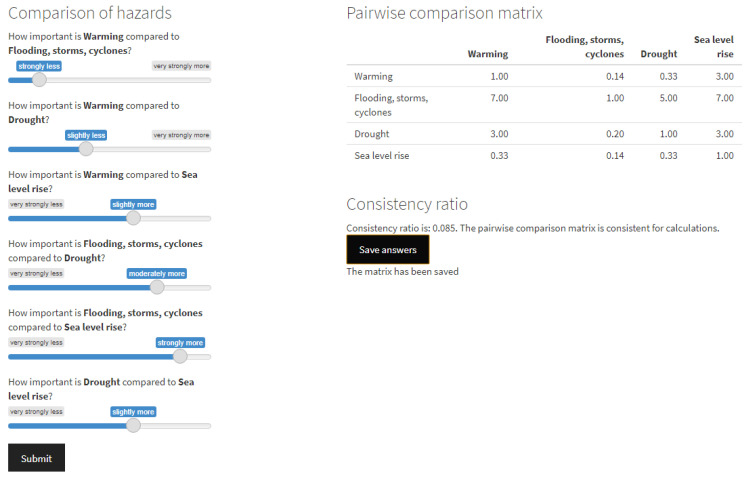
User interface showing the questions used to populate the pairwise comparison matrix using the example of climate hazards. The climate hazards (1) warming, (2) flooding, storms, and cyclones, (3) droughts, and (4) sea level rise were used for illustration purposes. After submitting all pairwise comparisons of climatic hazards (left panel), participants can see the pairwise comparison matrix (right panel) in real time and its associated consistency ratio. This step needs to be repeated for the other criteria, i.e., processes of transmission and health outcomes.

The climate sensitivity criteria defined in the previous step are then ranked following the analytical hierarchy process (AHP) methodology [19] by the workshop participants. In this process, experts are asked to compare the importance of each pair of criteria to determine their relative importance. A web-based user interface (https://qaohs.shinyapps.io/ptcsz/, accessed on 20 August 2024) was developed to facilitate the weighting of criteria by participants and automate the calculation of criteria weights using the R Shiny package (version 1.8.0). This interface allows participants to answer questions for the pairwise comparison of criteria selected in Activity 2 to populate a pairwise comparison matrix (right panel in Figure 3). The questions are structured in the format of “How important is *climate hazard 1* compared to *climate hazard 2*”. Participants can select the relative importance of one hazard over the other using a linguistic scale (left panel in Figure 3) with a slider widget on the user interface. This answer is then translated to the numerical intensity of importance [19], ranging from 1/9, representing *extremely less important*, to 9, representing *extremely more important* (Table 1).

Once all criteria have been compared against each other, the pairwise comparison matrix is shown to each participant (Figure 3). The interface ensures that the consistency of the pairwise comparison matrix is acceptable for each participant. The consistency of the matrix is assessed using the consistency ratio presented by Saaty [46] and calculated using the R package FuzzyAHP (version 0.9.5) [47]. If the pairwise comparison matrix of a participant is sufficiently consistent, i.e., with a consistency ratio of less than 0.10 [48], the matrix can then be saved or exported to a server. 

It is worth noting that the example provided serves as an initial adaptation or demonstration of how the interface could potentially appear. However, it is essential to emphasize that this demonstration is subject to modifications based on the outcomes derived from Activities 1 and 2, namely, the production of the list of zoonotic diseases earmarked for prioritization and the selection of climate sensitivity criteria. These results will play a pivotal role in shaping and customizing the interface to best suit the specifics of the country.

In addition to calculating weights according to the AHP methodology outlined by Saaty [19], the fuzzy weights using the triangular fuzzy number methodology [49] can be calculated using the geometric mean [50], as well as the crisp or defuzzified and normalized defuzzified weights. Examples of weights (based on the standard AHP), fuzzy weights, and defuzzified and normalized defuzzified weights calculated for different climate hazards based on the pairwise comparison matrix are presented in Table 2. Examining fuzzy weights allows us to assess variability in the relative importance of criteria.

This weighing process is repeated for the three sets of criteria, i.e., climate hazards, processes of transmission, and impacts on health outcomes. 

When all participants of a workshop have concluded the pairwise comparisons for all three sets of criteria, all matrices can be read and combined into a group matrix to calculate group weights for the three criteria using the geometric mean method [51].

#### 2.2.5. Activity 4: Development of Questions and Answers

Once the weights of criteria have been calculated, participants are required to refine questions from the preliminary list developed prior to the workshop and used to score the zoonoses identified in Activity 1. The questions are formulated by combining the three sets of criteria that were selected in Activity 2 and ranked in Activity 3. For instance, the question “Does warming impact on the evolution of pathogens and lead to an increase in mortality?” combines the climate hazard “warming”, the transmission process “evolution of pathogen”, and the health outcome “mortality”. A single weight for each question is then derived by averaging the weights of the criteria composing the question.

Participants are encouraged to select around 10–20 questions to limit the time needed to answer all questions for all diseases and aim for a balanced representation of each climate hazard across the questions. Possible answers to each question are then discussed and agreed on by the participants. To ensure consistency across all questions, answers should be multinomial and ordinal. For instance, for the question “Does warming impact on the evolution of pathogens that leads to increased transmission?”, the potential answers could include four options: (1) no change in transmission type, (2) increase transmission in animals only, (3) increase transmission in humans only, and (4) increase transmission in both animals and humans. Finally, given the range of expertise of the participants and the uncertainty related to the impacts of climate change on the transmission of zoonoses, participants are asked to provide their confidence level in each of their answers (low, medium, and high confidence). By providing a confidence level to the answer, the score for each question is adjusted based on the confidence level, thus providing an initial, lower, and upper score for each question.

#### 2.2.6. Activity 5: Scoring of Zoonoses

**Figure 4:**
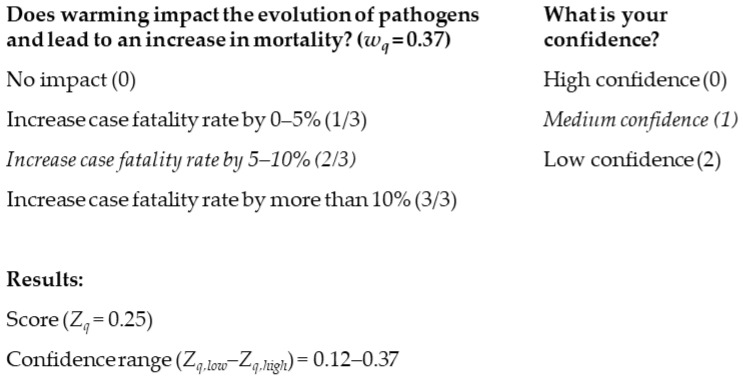
Example of answers and confidence levels for a given question and disease. The values attributed to each answer and confidence level are shown in brackets. The answer and confidence level selected by the participant in this example are shown in italics.

In the next step of the workshop, participants are asked to answer all questions previously agreed on in Activity 4 and provide their confidence level to all their answers using the web interface. Figure 4 shows an example of the score attributed to answers and confidence levels for the following question: “Does warming impact on the evolution of pathogens and lead to an increase in mortality?”. In this example, the selected answer is “Increase case fatality rate by 5–10%”, which has a value of 2 out of a maximum of 3, and the selected confidence level is medium. The initial score for this question *q* and disease *d* (*Z_q,d_*) is then calculated by multiplying the weight of the question determined in Activity 4 (*w_q_*) by the value of the answer (*V_q,d_*) over the maximum potential score for this question (*M_q_*) as follows:

(1) $$Z_{q,d}=w_{q}\times \frac{V_{q,d}}{M_{q}}$$

For this example, the resulting score would be 0.25 based on the weight of the question (0.37) and the value of the answer (2/3). To calculate the confidence range, the value of the confidence level (*C_q,d_*), in this case, 1, is subtracted by the value of the answer (*V_q,d_* = 2) to obtain the lower estimate ($Z_{low,q,d}$) as follows:

(2) $$Z_{low,q,d}=w_{q}\times \frac{V_{q,d}-C_{q,d}}{M_{q}}$$

Similarly, the confidence level attributed to the same question and disease is added to the answer value to obtain the higher estimate ($Z_{high,q,d}$) as follows:

(3) $$Z_{high,q,d}=w_{q}\times \frac{V_{q,d}+C_{q,d}}{M_{q}}$$

Therefore, the score given by this participant to this question and disease would range from 0.12 to 0.37 (Figure 4).

The final score of each disease (*Z_d_*) is then calculated by aggregating the score of all questions *q* over all participants *p* for each disease *d* as follows:

(4) $$Z_{d}=\sum_{p}^{P}\sum_{q}^{Q}Z_{d,p,q}$$

Similarly, the lower and higher score estimates based on the confidence levels are calculated by aggregating the lower ($Z_{low,d}$) and upper ($Z_{high,d}$) score estimates of all questions over all participants as follows:

(5) $$Z_{low,d}=\sum_{p}^{P}\sum_{q}^{Q}Z_{low,q,d}$$

(6) $$Z_{high,d}=\sum_{p}^{P}\sum_{q}^{Q}Z_{high,q,d}$$

**Figure 5:**
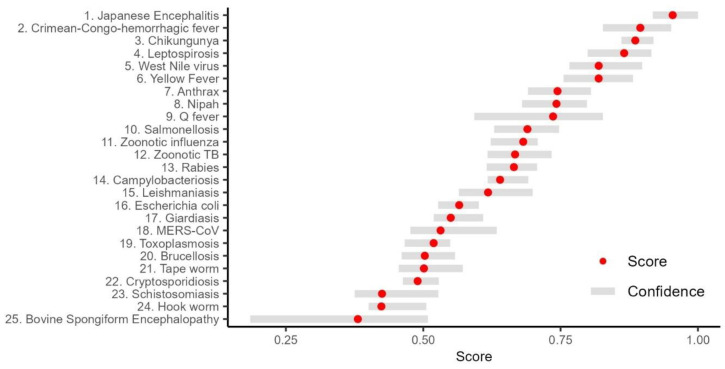
Example of the hypothetical scoring results of 25 zoonotic diseases for illustration purposes. The red points show the mean score, and the gray lines show the confidence range for each disease based on 10 hypothetical participants.

The final scores can be generated rapidly with the web interface and visualized as soon as all participants have answered all questions for all diseases. Final scores for the prioritization can be visualized with the initial score and a confidence range, as illustrated in Figure 5 in a fictitious example. In this example, 10 hypothetical experts participated in the workshop and answered 10 questions for each disease. According to this scenario, a total of 25 diseases were selected for scoring. The figure shows the initial estimate (red dot, *Z_d_*) and the confidence range (horizontal gray bar, *Z_low,d_* to *Z_high,d_*) over all participants and all questions, with a maximum normalized score of 1.

#### 2.2.7. Activity 6: Validation of the Priority List of Zoonoses

The results should be presented to the participants during the workshop to receive feedback on the priority list and the tool. Given the uncertain nature of climate change, surveillance data are likely to be lacking, and expert opinion and knowledge may not be sufficient to accurately estimate the future impacts of climate change on zoonotic disease prevalence or incidence in human and/or animal populations. However, discussion among the participants can help to reach a consensus around the final priority list and provide qualitative validation of the process.

## 3. Discussion

### 3.1. Criteria for Prioritizing Climate Sensitivity of a Zoonotic Disease

The definition of climate sensitivity in the context of zoonotic diseases requires a multifaceted approach given the complex causal pathway of relationships between biotic and abiotic factors. In this study, we developed a framework for defining criteria which capture three aspects of the epidemiological causal pathway through which climate change can affect human, animal, and environmental health through the modification of zoonotic disease transmission dynamics.

In the context of zoonotic disease transmission, we define climate sensitivity by combining the type of climate hazards, the biological mechanisms through which climate hazards impact transmission, and the associated human and animal health outcomes. This tridimensional approach is based on the findings of recent literature reviews [2,31,33,34,35] and allows a complete representation of the abiotic and biotic processes through which climate change can increase the vulnerability of the human and animal populations (including wildlife) to zoonoses. Additional climate hazards, e.g., ocean acidification, could be added to the list if deemed relevant to a country by local experts and expected to affect zoonotic disease transmission.

The second part of the climate sensitivity definition includes the biological mechanisms through which a particular climate hazard impacts zoonotic disease transmission. Increasing geographic exposure and contact between humans, livestock, wildlife, and vectors has been found to be a leading cause of zoonotic spillover [3,52]. For instance, clusters of Hendra virus transmission from Pteropodid bats to horses in subtropical Australia have been linked to unusually high residency periods of bats in agricultural areas as a result of nutritional stress from drought-induced food shortages [41]. In North America, the spatial distribution of the hispid cotton rat (*Sigmodon hispidus*) and the white-footed deer mouse (*Peromyscus leucopus*), hosts of the Black Creek Canal hantavirus, and the agents of Lyme disease, respectively, have both shifted northward as the result of more suitable annual temperature [53].

Change in host or vector population can be the result of optimal climatic conditions for pathogen reproduction. For instance, warming and intense precipitation have been linked to increased food availability, creating suitable habitat conditions and causing surges in rodent populations associated with cases of plague [53] and hantaviruses [54]. Temperature and humidity have also been found to significantly affect the survivorship of ticks [55,56]. 

The evolution of pathogens consists of the change in virulence, incubation period, or the survival rate of pathogens. The incubation periods of pathogens decrease, and their replication rates increase with elevated temperatures, expanding the pathogen load within vectors. For example, the rate of dengue virus replication in *Aedes aegypti* mosquitoes increases linearly with increasing temperature [57], and malaria parasites only develop in mosquito vectors within certain temperature ranges [58]. The extrinsic incubation period of dengue virus decreases as temperature increases [59], and variation in the extrinsic incubation period causes the greatest proportional increase in the risk of disease emergence at cooler temperatures where the mean incubation period is long and associated variation is large [60]. Conversely, climate factors can also negatively affect the survival of pathogens. For instance, the survival of *Y. pestis*, the etiological agent of plague, most efficiently develops a biofilm that causes infected fleas to increase feeding attempts and regurgitate *Y. pestis* back into host animals during feeding at temperatures lower than 28 °C [61,62]. As such, climatic factors influence not only the survival rate of vector populations but also pathogens.

Susceptibility to infections consists of the human capacity to cope with pathogens and can entail a change in access to healthcare or sanitation from destruction of the infrastructure or displacement of vulnerable populations [63] following extreme weather events, such as floods and storms [2,64]. Stress or undernutrition can also affect immunocompetence to disease [65,66]. Climate hazards can influence, to different degrees, one or more of these processes of zoonosis transmission.

The third component of the climate sensitivity definition is the public health outcomes that can result from the biological processes of transmission. This set of sub-criteria included disease severity, availability of controls, socio-economic impacts, and transmissibility, which have been traditionally used in other disease prioritization protocols, such as the OHZDP. Based on previous studies, each of these categories contains between four and seven indicators. For instance, the severity of the disease can be assessed with indicators including case fatality rate, disability weight, or prevalence of a disease. The ability to prevent and control a disease can be assessed with the existence of a response plan in place or the capacity for diagnosis and surveillance. The impact on transmissibility has previously been characterized by the type of transmission (e.g., transmission possible only between animals, between humans, or between both), the number of outbreaks that have been caused by a disease in the past, and the number of jurisdictions that are affected by a disease. Socio-economic impacts can be estimated with the impact of a disease on the trade of animal products, animal mortality, decrease in production, media, or public attention, and work and school absenteeism.

Importantly, the selection of criteria should be tailored to the jurisdiction of interest following deliberation among local experts (Section 2.2.3). For instance, the criteria “impact on trade” used to assess public health outcomes may be substantially more relevant in a country with large exports of animal products. 

Ensuring the consistency of answers for the ranking of criteria by local experts during Activity 3 of the workshop (ranking of the criteria) is of utmost importance, and workshop facilitators play a crucial role in this regard. To prevent any inconsistencies in answers, it is essential for facilitators to guide experts in providing consistent responses when comparing criteria. If needed, facilitators should advise experts to review their pairwise comparisons of criteria to address any discrepancies and ensure coherence in the ranking, which will enhance the reliability and validity of the relative importance of criteria.

Finally, certain criteria may exhibit close semantic relationships and interdependencies. For example, the criteria indicating socio-economic impacts “decrease in production” can be understood as a result of “animal mortality”. To offer a more comprehensive overview of the effects of climate change on the human and animal health sectors and avoid equivalent questions, workshop facilitators should assist experts in identifying and selecting criteria in a way that minimizes interdependencies and covers different One Health sectors.

### 3.2. Design of Questions and Answers to Be Used in the Ranking of CSZs

The development of questions for scoring diseases in Activity 4 should encompass the complex interplay between the environment, animals, and humans, requiring a formulation grounded in One Health principles. Given the multidimensional nature of the impacts of climate change on health outcomes, a substantial number of questions can be developed during a workshop. However, considering the typically extensive list of diseases to be scored by experts (approximately 40 to 60 diseases), it is imperative to limit the number of selected questions to allow participants sufficient time to answer all questions for each disease. Facilitators should be sure to include a representative number of sub-criteria, e.g., an equal distribution of climate hazards, across all questions to prevent biases toward any specific sub-criteria.

To enable uncertainty assessment within the priority list, it is advisable to design multinomial and ordinal responses to capture the granularity of confidence levels in the answers of expert participants. While binary options (e.g., yes or no) can be employed, a smaller selection of potential answers may result in broader ranges of uncertainty in the final scores. Therefore, ordinal response options should be preferred during Activity 4 of the workshop.

### 3.3. Biases and Interdisciplinary Challenges

The prioritization approach was based on expert opinion elicitation, which is in line with several previous studies on zoonotic disease prioritization [67]. Experts may have biases or subjective opinions that influence their rankings, leading to potential inaccuracies in the prioritization process. This bias could stem from personal experiences, professional affiliations, or preconceived notions about certain diseases. Further, the pool of available experts in some regions may have limited expertise in specific zoonotic diseases or climate-related impacts, which could affect the comprehensiveness and accuracy of the FAHP methodology and prioritization process. Integrating expertise from multiple disciplines, such as epidemiology, ecology, climatology, and veterinary science, is crucial for such a complex problem but can be challenging and may lead to differences in terminology, methodologies, and priorities among experts. Both expert biases and interdisciplinary challenges can lead to disagreement in the priority list. To mitigate these biases, it is critical to aim for a balanced representation of government and academic agencies across human, animal, and environmental health among voters. Further, workshop facilitators should aim to reach a consensus through deliberation when generating the list of diseases to be prioritized, the climate-sensitive criteria, and the questions and answers for disease scoring. Utilizing a workshop-based expert elicitation process for prioritizing climate-sensitive zoonotic diseases ensures that the resulting priority list is relevant, transparent, and trusted by policymakers and aligns with public health needs [68].

### 3.4. Quantifying Uncertainties in Zoonosis Prioritization

While several literature reviews have reported evidence on the biotic and abiotic processes through which climate hazards effect particular human or animal health outcomes, there is still considerable uncertainty with respect to the strength of the evidence across the multiple epidemiological zoonotic disease transmission pathways. To formally capture expert’s uncertainty levels throughout the prioritization protocol, our approach enables the quantification of two types of uncertainties: (1) uncertainty related to the ranking of climate sensitivity criteria, using the FAHP approach, and (2) uncertainty related to the scoring of zoonoses by considering the confidence in the answers provided by the workshop participants. This approach differs from previous studies that have omitted the assessment of uncertainty in the priority lists of zoonoses. Such assessment is important given the complex and unpredictable dynamics between the varied climate hazards, processes of transmission, and human health impacts of zoonotic diseases, making it challenging to accurately predict their future trajectory. 

By assessing uncertainty, decision makers can understand the range of potential prioritization outcomes, allowing for more informed and robust decisions. This information helps allocate resources effectively, ensuring that funding and attention are directed towards zoonoses with the highest and most likely potential impact on human and animal health. In addition, uncertainty assessment allows decision makers to identify areas where further research and data collection are needed. This information is critical for developing targeted interventions and strategies to address knowledge gaps and reduce uncertainties in future prioritization. Moreover, assessing uncertainty in expert elicitation fosters transparency and accountability in decision-making processes by acknowledging and communicating the limitations and potential biases of expert judgments. This encourages a more evidence-based approach and helps build public trust in disease prioritization efforts. Ultimately, by assessing uncertainty, decision makers can make more informed and effective decisions, leading to improved disease management, better resource allocation, and, ultimately, better health outcomes for human and animal populations.

### 3.5. Opportunities and Limitations of the Tool

We presented a web-based tool that streamlines the different steps to weigh criteria and score zoonoses. This online tool offers the advantage of limiting the risk of errors from data manipulation when using spreadsheets across multiple computer systems and compiling responses from multiple participants manually. While the tool is accessible on both computer and mobile platforms, it requires reliable internet connectivity and cloud storage to aggregate and report responses from all experts in real time. In some cases, the interface may need to be translated into the local language, which can extend the pre-workshop preparation time. Further applications of the tool and feedback from users will be required to improve the usability and flexibility to add and modify criteria; for instance, impacts on the health system and questions and answers for scoring.

### 3.6. Expanding Beyond Climate Sensitivity and Zoonoses

The framework presented in this paper is flexible and could be applied to a wider array of pathogens; for instance, non-zoonotic high-threat pathogens (e.g., Malaria, Dengue, and vaccine-preventable diseases). Additionally, while the focus here is on zoonoses’ sensitivity to climate change, the same process can be applied to other drivers of change, such as projected land use change, antimicrobial resistance, or changes in human behavior. To consider the impacts of other drivers of change, a reassessment of the linkages between the new driver (e.g., land use change) and the disease transmission mechanisms would be required. This would entail adapting the criteria, questions, and answers to better represent the impacts of the alternative drivers of disease emergence and spread. Considering both climate and land use change in the prioritization process would provide a holistic assessment of the future threats that zoonoses will pose to human and animal health.

## 4. Conclusions

In this paper, we presented a generalizable protocol to be used by countries to assess the impacts of climate change on the transmission dynamics of zoonoses and the resulting impacts on animal and human health. Based on this protocol, we delineated a standardized structured process to identify national priority zoonoses based on expert knowledge and the FAHP methodology. We also presented an innovative online user interface that facilitates ease of use and implementation of this process to inform complex One Health decision making in the context of climate-sensitive zoonotic disease prioritization. The availability of this tool contributes to delineating countries’ first steps to pandemic preparedness investments for cross-sectoral coordination and operational decision making in surveillance and control strategies given the significant current and future impacts of climate change on infectious disease transmission [2,3].

## Figures and Tables

**Table 1:** Scale of the relative importance used to weigh criteria. The linguistic scale is used in a questionnaire in the user interface and is translated to the relative importance to populate the pairwise comparison matrix.

Linguistic Scale	Intensity of Importance
Extremely less important	1/9
Strongly less important	1/7
Moderately less important	1/5
Slightly less important	1/3
Equally important	1
Slightly more important	3
Moderately more important	5
Strongly more important	7
Extremely more important	9

**Table 2:** Example of weights, fuzzy weights, and defuzzified and normalized defuzzified weights for four example climate hazards based on a pairwise comparison matrix shown in Figure 3.

Criteria	Weight	Fuzzy Weights	Defuzzified	Normalized Defuzzified
Warming	0.10	0.07, 0.10, 0.15	0.11	0.10
Flooding, storms, and cyclones	0.65	0.49, 0.65, 0.86	0.67	0.65
Drought	0.19	0.13, 0.19, 0.27	0.20	0.19
Sea level rise	0.06	0.04, 0.06, 0.09	0.06	0.06
